# Counting what matters: stillbirth estimates before 28 weeks of gestation in India

**DOI:** 10.1016/j.lansea.2026.100770

**Published:** 2026-04-17

**Authors:** Shiyam Sunder Tikmani, Sarah Saleem

**Affiliations:** Section of Population and Reproductive Health, Department of Community Health Sciences, Aga Khan University, Karachi, 74800, Pakistan

Stillbirth remains one of the most neglected outcomes in maternal and newborn health. Despite being considered as one of the sensitive markers of quality, equity, and health system responsiveness, estimating its burden in many low- and middle-income countries (LMICs) like India, remains challenging.^1^ This is largely because of inconsistent definitions, underreporting, and suboptimal capture in routine surveillance.^2^ In this context, Pandey and colleagues make an important contribution by estimating stillbirth not only at the conventional threshold of 28 weeks of gestation, but also at greater than or equal to 24 weeks and 20 weeks, using three rounds of National Family Health Survey (NFHS) data from 2005 to 2021. The study reports a stillbirth rate (SBR) of 12.8 per 1000 births at 28 weeks, increasing to 16.2 at 24 weeks and 22.0 at 20 weeks of gestation. The study further shows that in 2019–21, the highest proportion of stillbirths (42%) occurred between 20 and 28 weeks of gestation.^3^

These findings echo those of other published literature.^4^, ^5^, ^6^ Although the conventional threshold of greater than or equal to 28 weeks of gestation remains important, addressing the complete burden of stillbirth requires capturing earlier gestational losses for greater visibility, priority setting, and resource allocation. The district-level analysis in this study suggested moderate spatial clustering, with 12.2% of districts reporting an SBR of more than 20 per 1000 births. Though the temporal trend shows a decline in SBR between 2005–2006 and 2015–2016, the subsequent reduction appears modest. The risk factors associated with stillbirth included maternal illiteracy, short maternal stature, anemia, rural residence, caste disadvantage, and poor living conditions.^3^ These findings reinforce that stillbirth is not only an adverse pregnancy outcome, but a reflection of deeper social, nutritional, environmental, and health system inequity.^7^ Reducing this burden will require approaches beyond improvements in obstetric care alone, including sustained efforts to enhance women’s health and nutrition starting from preconception, reducing structural disadvantage, and expanding access to quality health services for the underserved population.

The study’s methodological limitations also deserve careful attention. Reconstructing stillbirths from the NFHS reproductive calendar, rather than relying on standard stillbirth variables, is a pragmatic response to data quality concerns, but it inevitably introduces uncertainty. The reported category of termination may include miscarriage, induced abortion, and stillbirth, while retrospective month-based reporting is prone to gestational age misclassification, rounding, and recall error. The data does not distinguish antepartum from intrapartum stillbirths and cannot capture proximal clinical causes. The identified risk factors should therefore be interpreted as associative rather than causal. These limitations warrant caution in the interpretation of the results, but do not diminish the study’s value in highlighting the likely scale and distribution of under ascertained stillbirth burden.

The policy implications of these findings are important. Although India has reduced stillbirth rates over time, a substantial burden before 28 weeks remains insufficiently addressed, with persistent regional hotspots and slow decline. Further progress will require more than surveillance alone. Stillbirth prevention depends on a continuum of quality care across pregnancy, childbirth, and the perinatal period. Although India has introduced sentinel stillbirth surveillance guidance, implementation remains uneven because of variable state capacity, incomplete capture of deaths outside facilities, inconsistent gestational age assessment, weak cause attribution, and limited use of surveillance data for local quality improvement.^5^ In addition, the availability and accessibility to emergency obstetric services remain essential to tackle the burden of intrapartum stillbirth.

Strengthening stillbirth surveillance in India will not be straightforward. If early gestation stillbirths remain largely invisible in measurement and policy discussion, prevention efforts will remain incomplete. A stronger surveillance approach, linked to improvement in antenatal and intrapartum care, referral pathways, and more equitable service delivery, while incorporating more proximal maternal, fetal, placental, and health system determinants, is the need of the hour.^8^

Pandey and colleagues remind us that what is not counted is often not seen, and what is not seen is less likely to be prevented. Their analysis underscores that stillbirths before 28 weeks deserve greater attention in surveillance and policy discussion.

## Contributors

SST wrote the first draft of the commentary. SS provided critical feedback on the commentary.

## Declaration of interests

Authors declare no conflict of interest.
